# Comparison of the early cardiac electromechanical remodeling following transcatheter and surgical secundum atrial septal defect closure in adults

**DOI:** 10.1186/s43044-021-00174-5

**Published:** 2021-06-10

**Authors:** Amr Mansour, Noha M. Gamal, M. Alaa Nady, Salwa R. Demitry, H. Shams-Eddin, Khaled M. El-maghraby

**Affiliations:** 1 Cardiology Department, Congenital and Structural Heart Disease Unit, Faculty of Medicine, Ain Shams University Hospitals, Cairo, Egypt; 2 Cardiology Department, Faculty of Medicine, Assiut University, Assiut, Egypt; 3 Cardiothoracic Surgery Department, Faculty of Medicine, Assiut University, Assiut, Egypt

**Keywords:** Transcatheter ASD closure, Surgical ASD closure, Electrical remodeling, Mechanical remodeling, Cardiac MRI

## Abstract

**Background:**

Secundum atrial septal defect (ASD) closure leads to electrical and mechanical remodeling that occurs early after shunt disappearance. The relationship between electromechanical remodeling using electrocardiogram (ECG) and cardiac magnetic resonance (CMR) after percutaneous and surgical closure has not yet been recorded in prospective studies.

**Objective:**

We thought to study right atrium (RA) and right ventricle (RV) changes by CMR 3 months after transcatheter and surgical closure and their comparison with electrical remodeling by ECG.

**Results:**

We prospectively evaluated 30 consecutive adult patients with isolated secundum ASD who were referred for (transcatheter and surgical) ASD closure.

There was significant reduction in all of the electrical parameters within the same group as compared to the baseline values, except P wave dispersion (Pd). (P max was 97.33 ± 16.67 (pre closure) to 76 ± 15.49 (post closure) in the device group and 97.33 ± 12.79 (preclosure) to 73.33 ± 16.32 (post closure) in the surgical group, QRS complex was 104 ± 18.82 (preclosure) to 80 ± 18.51 (post closure) in the device group and 106.67 ± 14.47 (preclosure) to 86.67 ± 17.99 (post closure) in the surgical group. QTc maximum was 478.53 ± 36.79 (preclosure) to 412.53 ± 38.03 (post closure) in the device group and 470.53 ± 65.70 (preclosure) to 405.93 ± 63.08 (post closure) in the surgical group, and QTc dispersion was 70.33 ± 24.04 (preclosure) to 60.26 ± 28.56 (post closure) in the device group and 80.73 ± 30.38 (preclosure) to 60.27 ± 28.57 (post closure) in the surgical group).There was no significant difference between two groups indicating that transcatheter and surgical closure had led to equivalent value of electrical remodeling.

In CMR study, we measured RA maximal volume and right ventricle end diastolic volume (RVEDV), RA maximal volume decreased significantly as compared to the base line values post closure in both groups (P value < 0.001). The reduction in RA max volume was more in the transcatheter closure group; however, this difference was not statistically significant when compared with the surgical arm (*P* value = 0.5).RVEDV decreased significantly in both groups as compared to the baseline values (*P* value < 0.001). Transcatheter closure resulted in more significant reduction in the RVEDV than the surgical closure (*P* value = 0.03).

**Conclusion:**

Our study showed early significant electromechanical reverse remodeling in most of the study parameters from the baseline values after ASD closure. We found no significant differences in all of the electrical and RA mechanical remodeling parameters with significantly better mechanical remodeling of RV in the device group.

## Background

ASD is among the most common acyanotic congenital cardiac lesion [1]. Secundum ASD contributes to RA and RV volume overload, and chronic volume overload leads to right sided heart failure, pulmonary hypertension, and atrial arrythmias [2]. ASD closure is assumed to reverse RA and RV changes leading to electrical and mechanical remodeling. Although surgical closure of an ASD is a frequently offered low-risk procedure, it is associated with some morbidities including post pericardiotomy syndrome, arrhythmia, and scar formation [3]. Transcatheter closure of ASD has become an effective alternative to secundum ASD closure, avoiding complications of surgical closure [4]. We used CMR for assessment and evaluation of the RV and RA volumes as it is now considered the gold standard tool [5].

### Aim

We thought to study RA and RV changes by CMR 3 months after transcatheter and surgical closure and their comparison with electrical remodeling by ECG.

## Methods

This study which is longitudinal observation study was approved by our institutional and local review board, and written informed consent was obtained from all of the patients enrolled in this study. Thirty consecutive patients were included with isolated secundum ASD, 15 patients underwent successful transcatheter ASD device closure and 15 had surgical closure. All patients were subjected to full history, proper physical examination, 12 leads ECG and CMR before the ASD closure, and 3 months later.

Patients with secundum ASD and left to right shunt, sinus rhythm with increased RV volume load (QP/QS ratio > 1.5 and/or RV dilation) were included. Patients with secundum ASD and associated coronary artery disease or other congenital heart disease were excluded. Patients with pulmonary arterial hypertension and pulmonary vascular resistance (PVR) > 5 woods units (this according to the ESC guidelines of GUCH 2010 which considered ASD closure if PVR ≥ 5 as class IIB indication for closure which was also stated in the ESC guidelines 2020 as class III in PVR > 5 despite targeted PAH treatment) and partial anomalous pulmonary venous return were also excluded.

All patients were presented in the joint cardio-surgical meeting with the cardiology and cardiothoracic surgery. Patients with evidence of elevated pulmonary artery pressure or patients for whom non-invasive assessment of the pulmonary artery pressure was impossible or inconclusive underwent an invasive haemodynamic study as well as assessment of the pulmonary artery pressure and pulmonary vascular resistance. So, not all the surgical study cohorts underwent invasive pulmonary artery pressure evaluation. All patients who underwent transcatheter closure had assessment of the pulmonary vascular resistance.

A custom-made sheet was made for all the patients, 12 leads surface ECG as well as CMR assessment were obtained preclosure and 3 months after closure of the ASD.

### Electrocardiographic study

ECG machine used was electrocardiograph ECG-2250-Nihon Kohden.

**Fig. 1:**
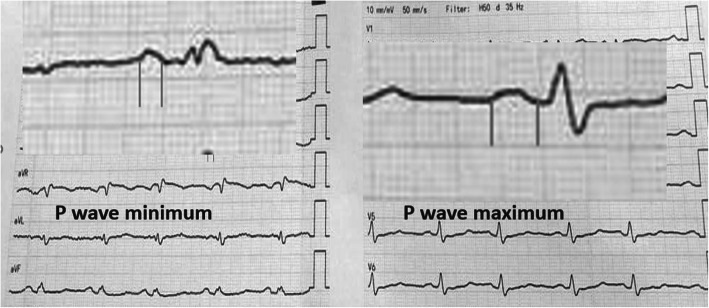
12 leads ECG in patient with secundum ASD showed P wave duration (Pmax, P min) measurement

A 12-leads ECG was recorded at a speed of 50 mm/s and an amplitude of 1 mV/cm before and 3 months after ASD closure for evaluation of the following electrical parameters:

P wave duration is defined as the distance between the junction of baseline with the point of the earliest and point of latest P wave activity. The longest P wave duration was noted as P wave maximum (P max) and the shortest duration as P wave minimum (P min) in any of the 12 ECG leads (Fig. 1) [6].

P wave dispersion (Pd) is defined as the difference between the maximum and the minimum P-wave duration recorded [6]. We selected P wave duration and P wave dispersion as they are strong surrogates for prediction of atrial arrythmia.

**Fig. 2:**
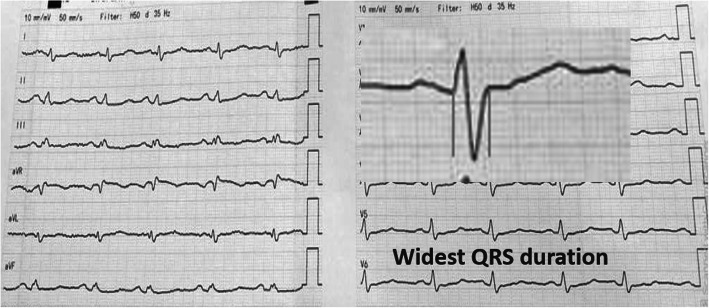
12 leads ECG in patient with secundum ASD showed QRS duration measurement

QRS duration is defined as the widest QRS complex duration in any lead as measured from the first deflection to the last deflection crossing the isoelectric line (Fig. 2) [7].

**Fig. 3:**
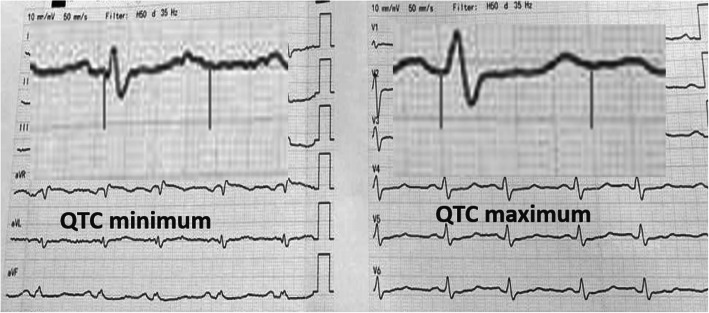
12 leads ECG in patient with secundum ASD showed QTc interval measurement

QTc interval is defined as the interval between the beginning of the QRS complex and the end of the T wave and corrected for patients’ heart rate using Bazett formula (Fig. 3) [8].

QT dispersion (QTD) is defined as the difference between the longest (QTc max) and the shortest (QTc min) QTc intervals within a 12-leads ECG [8].

### Echocardiography either transthoracic (TTE) or transoesophageal (TEE)

TTE was used to asses mean pulmonary artery pressure (MPAP) from the peak PR Doppler signal using the following formula: MPAP = 4( PR peak velocity)^2^ + RAP [9]. TEE was used to assess ASD size and its rims, and exclude anomalous pulmonary venous drainage.

### Cardiac magnetic resonance assessment

All CMR studies were performed with subjects in the supine head first position. Using a 1.5 Tesla MRI scanner (Ingenia Philips) using a surface cardiac coil and cardiac imaging software. Scanning was performed with ECG gating during end-expiratory breath-holding phase. Steady-State Precession (SSFP) ECG gated sequences were obtained retrospectively (Image matrix 256 × 150, field of view 380 mm, repetition time 52.05 ms, echo time 1.74 ms, and flip angle 70°) [10].

MRI was used to assess ASD size, rims and shunt fraction (QP\QS). For the ventricular image set, the stack of cine SSFP images were acquired in the short axis view from the level of the mitral valve to the left ventricular apex with 8 to 12 slice thickness, and measurements were indexed to the patient’s body surface area (BSA) [11]. Analysis of the RV was performed on per slice basis by manual contouring of the endocardial and epicardial borders. Volumes were calculated based on the Simpson’s method [12]. We included the trabeculations and papillary muscles as part of the RV volume [13]

RA maximum volume was measured using the biplane area method technique in 4 and 2 chamber views [14]. The maximal RA volume was traced during ventricular systole and was defined as last cine image before opening of the tricuspid valve [14]. The RA appendage was included in the RA volume while the inferior and superior vena cava were excluded [14].

### ASD closure

Percutaneous transcatheter ASD closure was performed when the anatomical characteristics and rims were adequate under general anesthesia with fluoroscopic and TEE guidance. Heparin (100 IU/kg) was given in every case. To avoid over sizing, a 24- or 34-mm sizing balloon (AGA Medical Corp.) was used to measure the diameter of the defect, and the stop-flow method balloon sizing was done with stop flow echo technique and fluoroscopic measurement was used for measurement in every patient. We did two cases through LUPV deployment technique and one through RUPV deployment technique. Balloon-assisted method (BAT) was done in one patient with absent aortic rim and with the help of a contralateral venous sheath.

The procedure was done using Amplatzer septal occluder (ASO) device, its sizes were 24 ± 6.6 mm ranging from 11 to 38 mm.

Surgical closure was done for patients with defects that were not suitable for transcatheter closure (with inadequate rims) by means of patch technique.

### Statistical analysis

Data was collected and analysed using SPSS (Statistical Package for Social Science, version 25, IBM, and Armonk, NY). Numerical data was expressed in the form of mean ± SD, while categorical data was expressed in the form of frequency and percentage.

Categorical data of different groups was compared by chi-square test while numerical data of both groups was compared by Mann-Whitney U test. Baseline and follow up data of the same group was compared by Wilcoxon signed-rank test. Percentage of change between baseline and follow-up data was calculated by the following equation; percentage of change = ((follow up-baseline data)/baseline data)) × 100.

Level of confidence was kept at 95% and hence, *P* value was considered to be significant if < 0.05.

## Results

### Demographic and clinical data of the study groups

The mean age of patients who underwent device closure was 33.73 ± 13.06 years while the mean age of those who underwent surgical closure was 35.33 ± 15.18 years with insignificant difference between both groups (*P* = 0.75) (Table 1). In both groups, majority (66.7%) of patients were females and less than 40 years old. There were insignificant differences between both groups as regards HR and BMI. All patients were neither DM nor HTN.

**Table 1:** Demographic date of studied patients

	Group 1 (device closure) (n = 15)	Group 2 (surgical closure) (n = 15)	*P* value
Age (years)	33.73 ± 13.06	35.33 ± 15.18	0.75
Sex			0.65
Female	10 (66.7%)	10 (66.7%)	
Male	5 (33.3%)	5 (33.3%)	
BMI (kg/m^2^)	24.78 ± 2.94	24.44 ± 4.02	0.79
Heart rate (bpm)	92 ± 10.14	93 ± 9.59	0.78

Data expressed as frequency (percentage), mean (SD). *P* value was significant if < 0.05

### Assessment of the electrical remodeling parameters

Both groups showed significant reduction in all ECG parameters—apart from P wave dispersion—after ASD closure when compared to the baseline values (Table 2). However, when we compared between the two methods of closure, we did not find significant difference between them as regards the electrical parameters (Table 3).

**Table 2:** ECG parameters before and after ASD closure in device and surgical group

	Group 1 (device closure)			Group 2 (surgical closure)		
	Before	After	P value	Before	After	P value
HR (bpm)	92 ± 10.14	78 ± 6.76	< 0.001	93 ± 9.59	78.67 ± 11.87	< 0.001
P max(ms)	97.33 ± 16.67	76 ± 15.49	< 0.001	97.33 ± 12.79	73.33 ± 16.32	< 0.001
P min(ms)	68 ± 16.56	48.67 ± 12.45	< 0.001	66.67 ± 12.34	45.33 ± 11.87	< 0.001
Pd (ms)	29.33 ± 10.32	27.33 ± 9.61	0.42	32 ± 12.64	28 ± 10.14	0.08
QRS (ms)	104 ± 18.82	80 ± 18.51	< 0.001	106.67 ± 14.47	86.67 ± 17.99	< 0.001
QTc max(ms)	478.53 ± 36.79	412.53 ± 38.03	< 0.001	470.53 ± 65.70	405.93 ± 63.08	< 0.001
QTD (ms)	70.33 ± 24.04	60.26 ± 28.56	0.003	80.73 ± 30.38	60.27 ± 28.57	0.001

*P max* P wave maximum, *P min* P wave minimum, *Pd* P wave dispersion, *QTD* QTdispersion. Data expressed as frequency (percentage), mean (SD). *P* value was significant if < 0.05

**Table 3:** ECG parameters comparing both groups

	Group 1 (device closure) (*n* = 15)	Group 2 (surgical closure) (*n* = 15)	*P* value
Heart rate (bpm)			
Baseline	92 ± 10.14	93 ± 9.59	0.78
Post closure	78 ± 6.76	78.67 ± 11.87	0.85
Percentage of change	(−) 14.68 ± 8.07	(−) 15.40 ± 8.89	0.81
P wave maximum (ms)			
Baseline	97.33 ± 16.67	97.33 ± 12.79	0.99
Post closure	76 ± 15.49	73.33 ± 16.32	0.65
Percentage of change	(−) 21.67 ± 9.51	(−) 24.88 ± 10.41	0.38
P wave minimum (ms)			
Baseline	68 ± 16.56	66.67 ± 12.34	0.80
Post closure	48.67 ± 12.45	45.33 ± 11.87	0.45
Percentage of change	(−) 27.89 ± 9.90	(−) 31.11 ± 14.93	0.49
P wave dispersion (ms)			
Baseline	29.33 ± 10.32	32 ± 12.64	0.53
Post closure	27.33 ± 9.61	28 ± 10.14	0.85
Percentage of change	(-) 3.67 ± 0.36	(−) 8.88 ± 3.78	0.47
QRS complex (ms)			
Baseline	104 ± 18.82	106.67 ± 14.47	0.66
Post closure	80 ± 18.51	86.67 ± 17.99	0.32
Percentage of change	(-) 22.84 ± 11.64	(-) 19.11 ± 10.25	0.36
QT maximum (ms)			
Baseline	478.53 ± 36.79	470.53 ± 65.70	0.68
Post closure	412.53 ± 38.03	405.93 ± 63.08	0.73
Percentage of change	(−) 13.63 ± 6.98	(−) 13.67 ± 6.98	0.98
QT minimum (ms)			
Baseline	405.73 ± 41.27	389.80 ± 63.74	0.42
Post closure	345.53 ± 71.84	345.67 ± 56.40	0.99
Percentage of change	(−) 14.99 ± 5.88	(−) 11.11 ± 6.29	0.38
QT dispersion (ms)			
Baseline	70.33 ± 24.04	80.73 ± 30.38	0.30
Post closure	60.26 ± 28.56	60.27 ± 28.57	0.66
Percentage of change	(−) 19.33 ± 11.28	(−) 24.98 ± 10.16	0.42

*P max* P wave maximum, *P min* P wave minimum, *Pd* P wave dispersion, *QTD* QTdispersion. Data expressed as frequency (percentage), mean (SD). *P* value was significant if < 0.05

### ASD diameter, rims assessment, MPAP, and Shunt fraction (QP/QS)

**Fig. 4:**
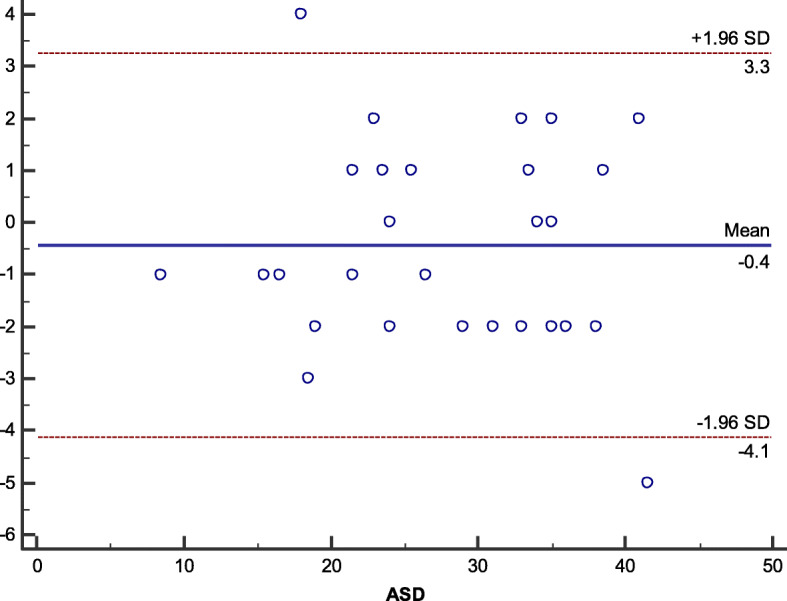
Bland-Altman plot comparing ASD with TEE and MRI showed strong degree of agreement (0.96)

Assessment of ASD diameter and rims using TEE and MRI showed strong degree of agreement (0.96), using Bland-Altman plot as illustrated in Fig. 4.

Shunt fraction assessed by CMR showed insignificant statistical difference between both groups (Table 5).

Patients underwent device closure had significantly lower MPAP (18.13 ± 7.21 vs. 23.93 ± 6.63 (mmHg); *P* = 0.03) in comparison to surgical device after closure with statistically insignificant difference pre-closure (Tables 4 and 5).

**Table 4:** PAP in device and surgical group

	Group 1 (device closure) (*n* = 15)			Group 2 (surgical closure) (*n* = 15)		
	Before closure	After closure	P value	Before closure	After closure	*P* value
MPAP (mmHg)	27.93 ± 5.18	18.13 ± 7.21	0.001	30.67 ± 5.30	23.93 ± 6.63	0.001

Data expressed as frequency (percentage), mean (SD). *P* value was significant if < 0.05

PAP: pulmonary artery pressure

**Table 5:** PAP and shunt fraction (QP/QS) in both groups

	Device closure (*n* = 15)	Surgical closure (*n* = 15)	*P* value
MPAP (mmHg)			
Baseline	27.93 ± 5.18	30.67 ± 5.30	0.16
Post-closure	18.13 ± 7.21	23.93 ± 6.63	0.03
QP/QS ratio			
Before closure	2.03 ± 0.25	2.23 ± 0.40	0.11
After closure	0.95 ± 0.13	1.04 ± 0.09	0.04

Data expressed as frequency (percentage), mean (SD). *P* value was significant if < 0.05

*PAP* pulmonary artery pressure, *QP\QS* shunt fraction

### Assessment of the mechanical remodeling by CMR

#### RA measurements

RA maximal volume was significantly reduced in both groups when compared with the baseline values (Table 6). However, there was no significant difference between both groups (Table 7).

**Table 6:** Parameters of RA and RV in device and surgical groups

	Group 1 (device closure) (*n* = 15)			Group 2 (surgical closure) (*n* = 15)		
	Before closure	After closure	*P* value	Before closure	After closure	*P* value
RA maximal volume (mm)	74.53 ± 23.98	46.88 ± 12.77	< 0.001	81.90 ± 30.07	55.38 ± 18.03	< 0.001
RVEDV (mm)	133.33 ± 25.26	87.10 ± 14.08	< 0.001	152.33 ± 52.36	106.60 ± 31.21	< 0.001

RA: right atrium, RVEDV: right ventricle end diastolic volume. Data expressed as frequency (percentage), mean (SD). *P* value was significant if < 0.05

**Table 7:** Parameters of RA and RV in comparison among two groups

	Group 1 (device closure) (*n* = 15)	Group 2 (surgical closure) (*n* = 15)	*P* value
RA maximum volume (ml/mm^2^)			
Before closure	74.53 ± 23.98	81.90 ± 30.07	0.46
After closure	46.88 ± 12.77	55.38 ± 18.03	0.14
Percentage of change	(−) 34.38 ± 17.47	(−) 30.15 ± 17.11	0.50
RV-EDV (ml/mm^2^)			
Before closure	133.33 ± 25.26	152.33 ± 52.36	0.21
After closure	87.10 ± 14.08	106.60 ± 31.21	0.03
Percentage of change	(-) 32.60 ± 13.55	(−) 27.20 ± 15.07	0.31

*RA* right atrium, *RVEDV* right ventricle end diastolic volume. Data expressed as frequency (percentage), mean (SD). *P* value was significant if < 0.05

#### RV measurements

RVEDV showed significant changes from the baseline measurements in both groups after ASD closure (Table 6). Comparing between both groups showed significant difference between them with more reduction in the transcatheter arm (Table 7).

## Discussion

We included a total number of thirty adult patients in a prospective study to compare short term electrical and mechanical remodeling parameters after transcatheter and surgical ASD closure. Each group contained 15 age-, sex-, and BSA-matched patients.

The main purpose of ASD closure is the elimination of shunt with excess right heart volume. In our cohort, ASD surgical closure resulted in both electrical and mechanical remodeling of the same as percutaneous closure with better RV remodeling results in the device group. In early series, RV enlargement persisted in almost two thirds of both pediatric and adult patients over a mid-term echocardiography follow-up [15].

CMR has been shown to be both reliable and reproducible in terms of quantitative RV assessment. The combination of a time-resolved 3D data set, clear distinction between the blood pool and the myocardium, and high spatial and temporal resolutions allow for accurate measurements of the RV, regardless of its morphology or orientation within the thorax, and without geometric assumptions [16].

### Atrial electrical remodeling

In our cohort, there was a reduction in the electrical parameters—resting heart rate, Pd, max, and min P wave duration after ASD closure in comparison to the baseline measurements in both groups, all showed statistically significant P value < 0.05, with exception of Pd which was statistically insignificant with P value = 0.4 in group 1 and 0.08 in group 2. Our results agreed with the study conducted by Kamphuis et al. [7], who found that the reduction in the resting heart rate after ASD closure was due to decrease in RA size, and this led to deactivation of the Bainbridge reflex [17] and decrease stretching of the pacemaker tissue in the sinus node [18].

The change of electrical remodeling parameters—Pmax, P min, and Pd—from the baseline measurements after ASD closure was more in the surgical group than in the transcatheter group; however, the difference between the two groups did not reach a statistical significance P value > 0.05. This indicates that transcatheter and surgical ASD closure had led to equivalent degrees of electrical remodeling with no superiority over each other. This partially agree with the results of the two studies conducted by Baspinar et al. [19] and Muzaffer et al. [20], who reported no difference in the P max values measured early after transcatheter and surgical ASD closure. In contrary to our study, they demonstrated significant reduction in the Pd measurements in the surgical arm in comparison to the transcatheter arm. This effect was clarified by the bulk of the device. Also, Paç et al. [6] found in their study that the atrial disc diameter and device sizes were the strongest correlates with the change in the Pd values. The discrepancy between our findings and the studies described above may be explained by the difference in the mean age of the research cohorts. The Pac et al. study included younger patients (mean age = 7.2 ± 3.3), and the mean age of our cohort was = 34.53 ± 14.12. This observation may emphasize the importance of the duration of shunt as an important factor affecting the degree of electrical remodeling, where longer duration of shunt may result in less early reversibility of Pd values with resultant prolongation of atrial refractoriness heterogenicity.

### Ventricular electrical remodeling

In our study, there was significant shortening in the QRS complex duration, QTc max, and QTD after ASD closure as compared to the baseline measurements in both groups. However, there was no significant difference between the two groups. This agree with Veldtman et al. [21] and Gatzoulis et al. [22], who showed significant reduction in the QRS complex duration after ASD device closure; this was explained by the reduction of RV volume overload, geometrical remodeling, and improvement of pulmonary artery pressure with partial reversal of right bundle branch block. Also, Rücklová et al. [23] found significant reduction in the QT dispersion 1 month after the intervention either transcatheter or surgical closure. We did not find superiorly of any method of closure on the ventricular electrical remodeling.

### Mechanical remodeling by CMR

#### Atrial measurements

**Fig. 5:**
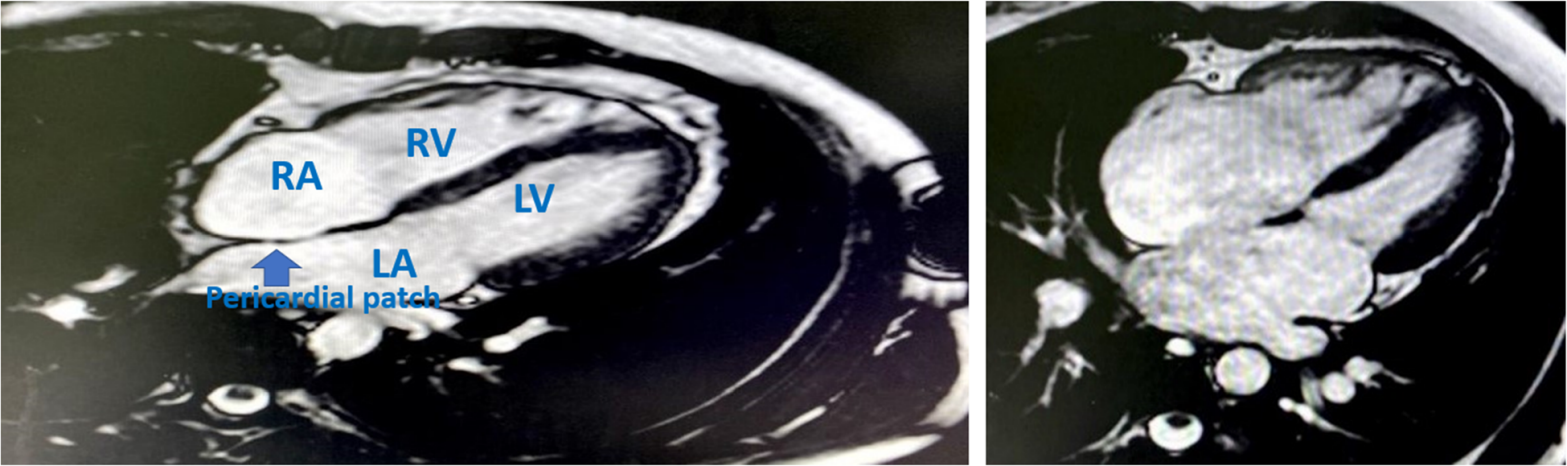
Cine SSFP 4 chamber MRI view in surgically closed case by conventional method with revolution of RA and RV volumes

**Fig. 6:**
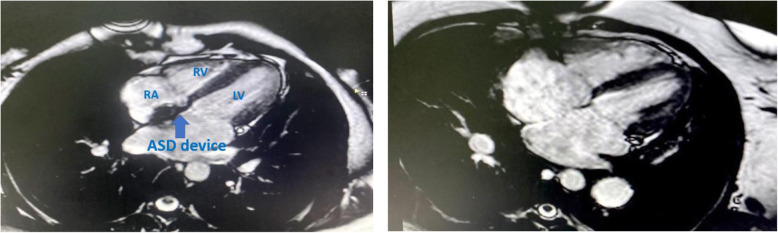
Cine SSFP 4 chamber MRI view in transcatheter closed case by ASO

Our study showed significant reduction in the indexed RA maximal volume and indexed RVEDV after ASD closure in both groups as compared to baseline measurements (Figs. 5 and 6). There was tendency towards more reverse remodeling in the indexed RA maximal volume in the device group; however, this was not statistically significant when compared to the surgical group.

#### Ventricular remodeling

Transcatheter ASD closure had led to more reverse remodeling in the indexed RVEDV measurements when compared to the surgical arm (Fig. 5). This agreed with Pascotto et al. [24] who reported failure of the surgical ASD closure to completely revert the right ventricular chamber overload, they explained this by either functional changes secondary to the effects of the cardio-pulmonary bypass technique or cardiac geometric alteration resulting from opening of the pericardial sac. Our results disagree with the study performed by Foo et al. [25] who found no difference between the surgical and device closure groups. This can be explained by the difference of modalities used for analysis of the RV volumes. In our study, we used the CMR which is considered as the gold standard for RV volumetric assessment, while Foo et al. used (2D) echocardiography which relies heavily on geometrical assumption.

## Limitations and recommendations

- Our study population was relatively small in number with short to intermediate term follow-up. Larger sample size and longer-term follow-up are needed to correlate this finding with clinical outcome and incidence of arrhythmia.

## Conclusion

- Transcatheter and surgical ASD closure have led to equivalent degrees of atrial and ventricular electrical remodeling, atrial mechanical remodeling, and better ventricular mechanical remodeling as evidenced by CMR.
- Pd values did not change significantly from the baseline despite significant mechanical changes after both methods of ASD closure.
- Transcatheter ASD closure had led to better RV mechanical remodeling than surgical closure.

## Data Availability

The datasets used and/or analyzed during the current study are available from the corresponding author on reasonable request.

## Abbreviations

ASD: Atrial septal defect
ECG: Electrocardiogram
CMR: Cardiac magnetic resonance
Pd: P wave dispersion
RA: Right atrium
RVEDV: Right ventricle end diastolic volume
PAH: Pulmonary arterial hypertension
PVR: Pulmonary vascular resistance
BSA: Body surface area
TEE: Transoesophageal echocardiography
P max: P wave maximum
P min: P wave minimum
Pd: P wave dispersion
QTD: QT dispersion
SSFP: Steady-state precession
TTE: Transthoracic echocardiography
ASO: Amplatzer septal occluder
